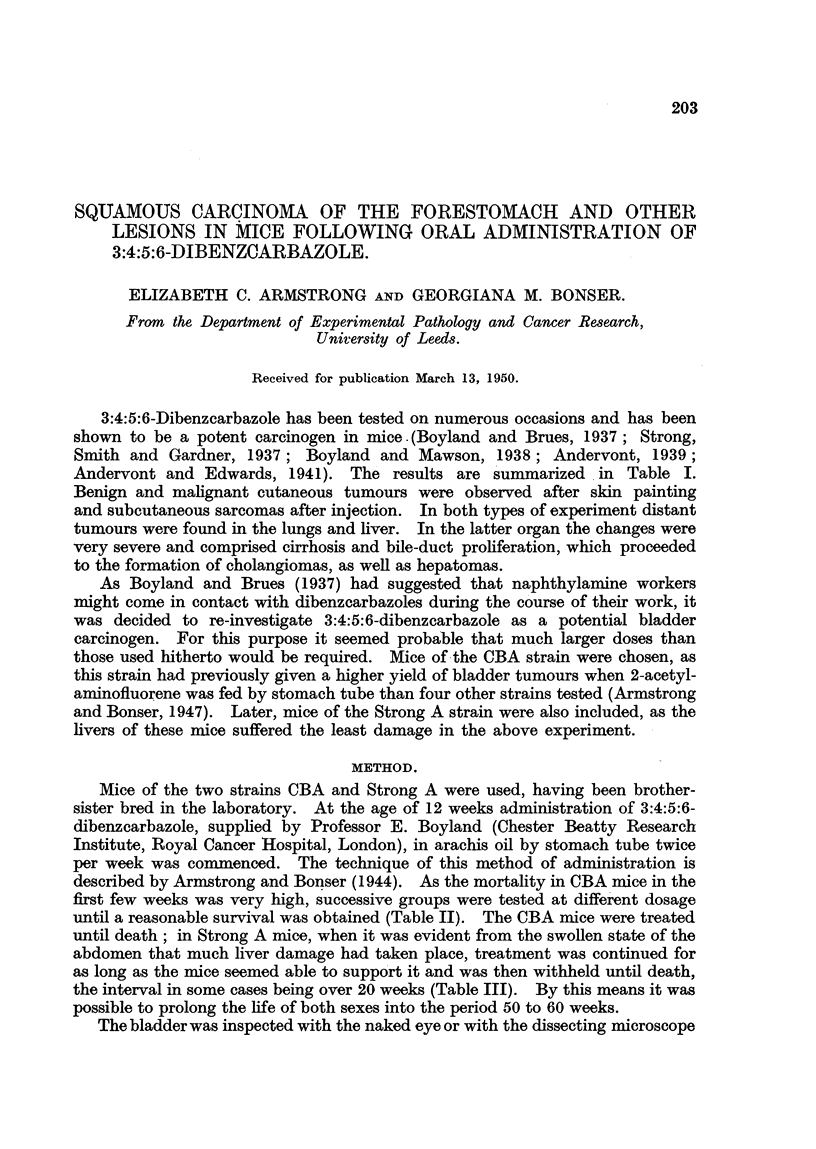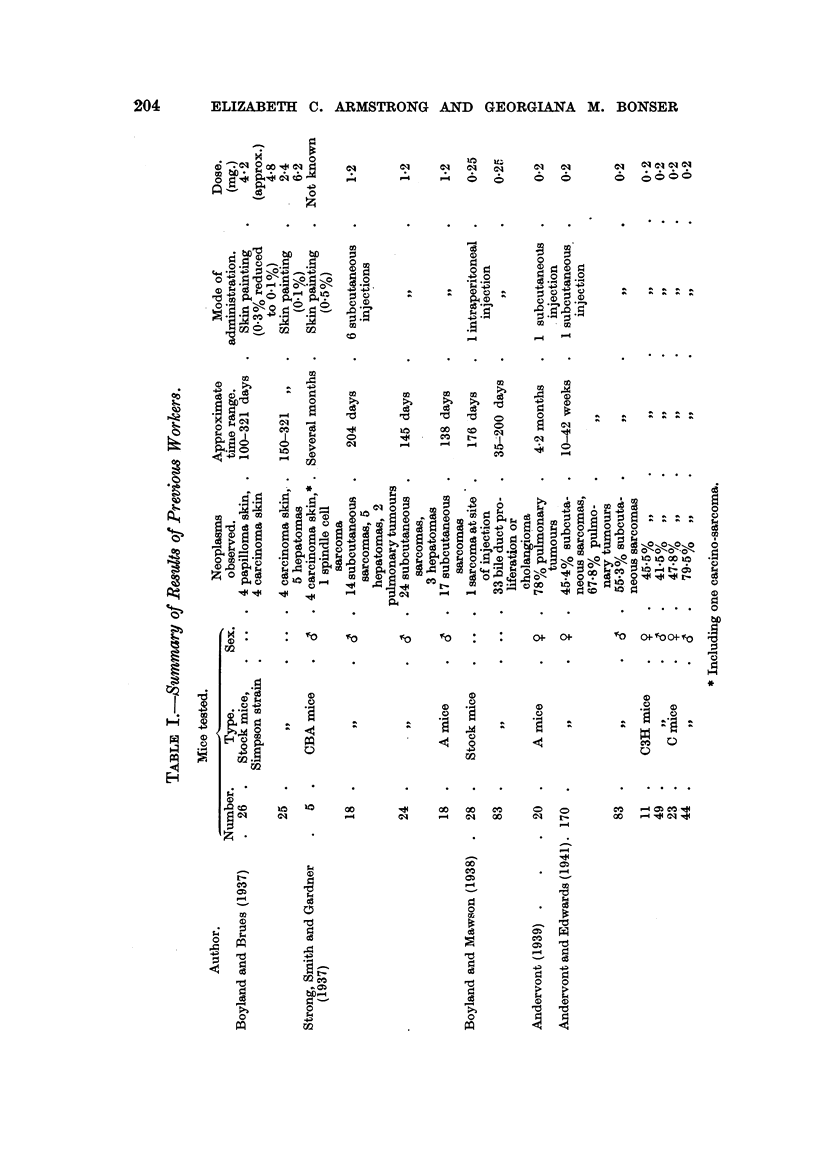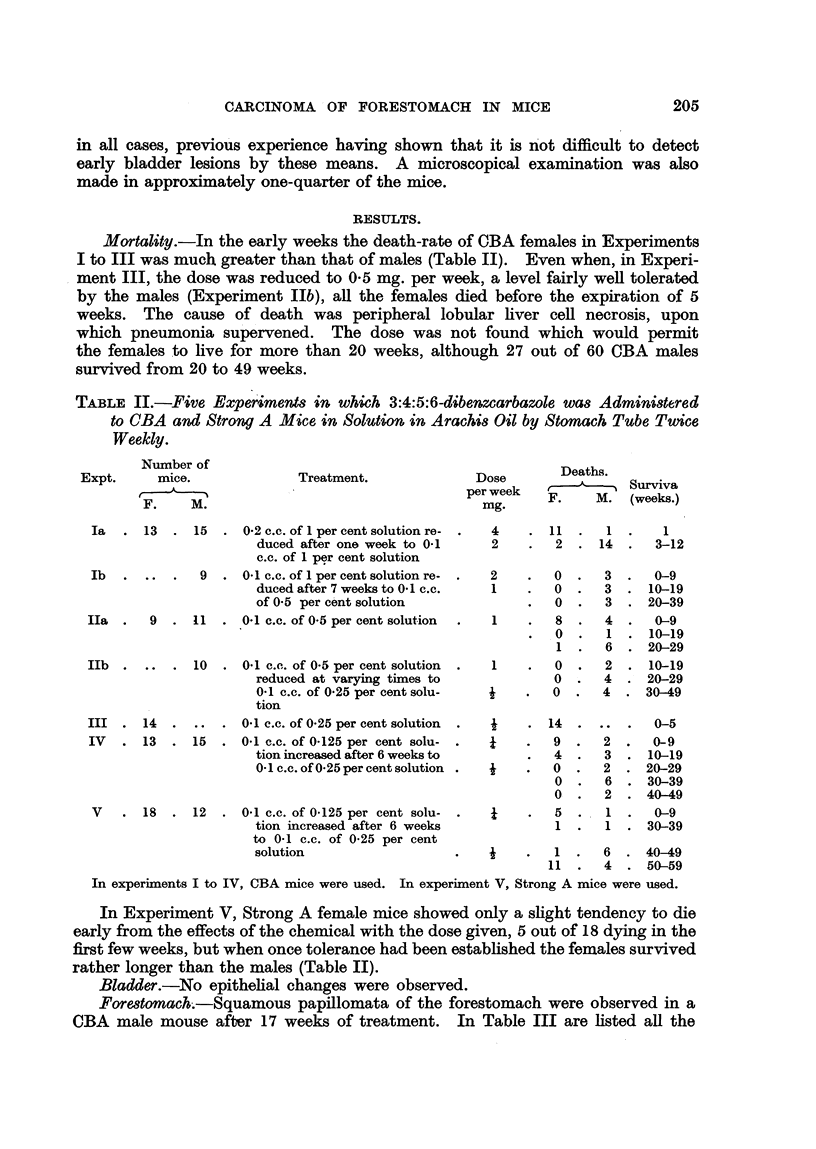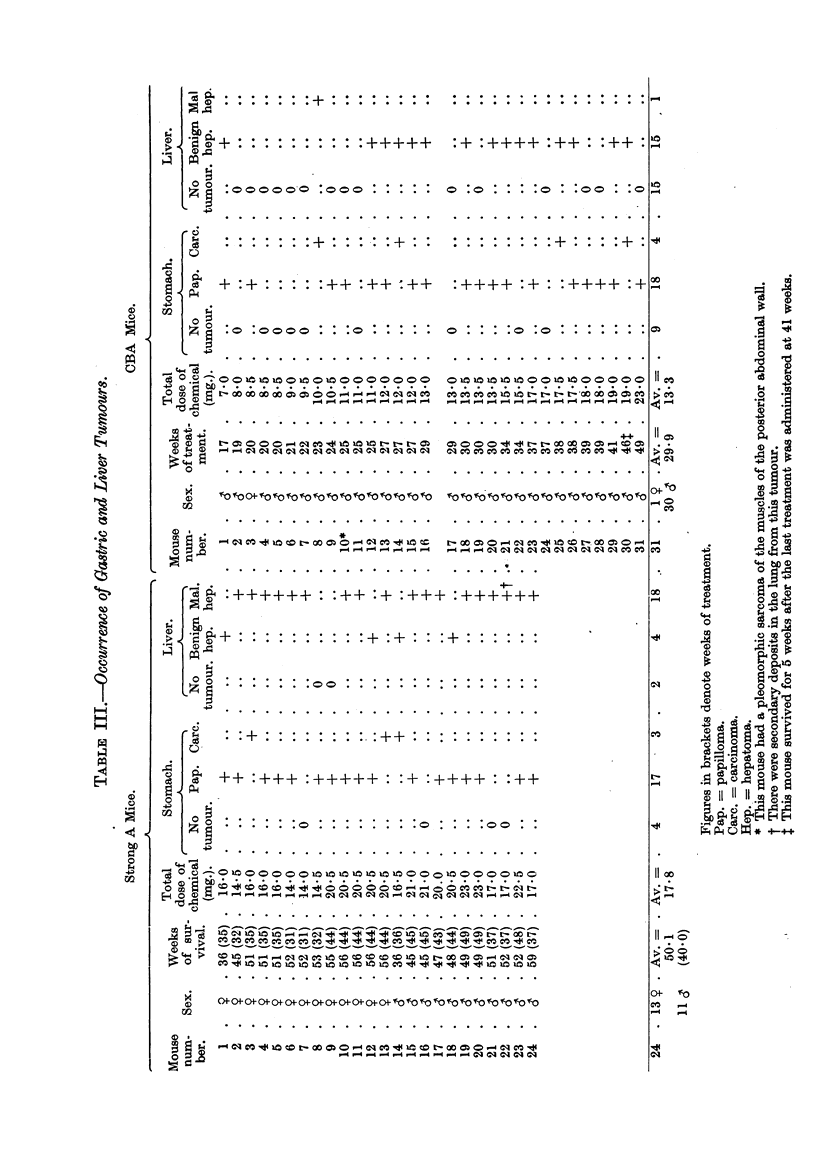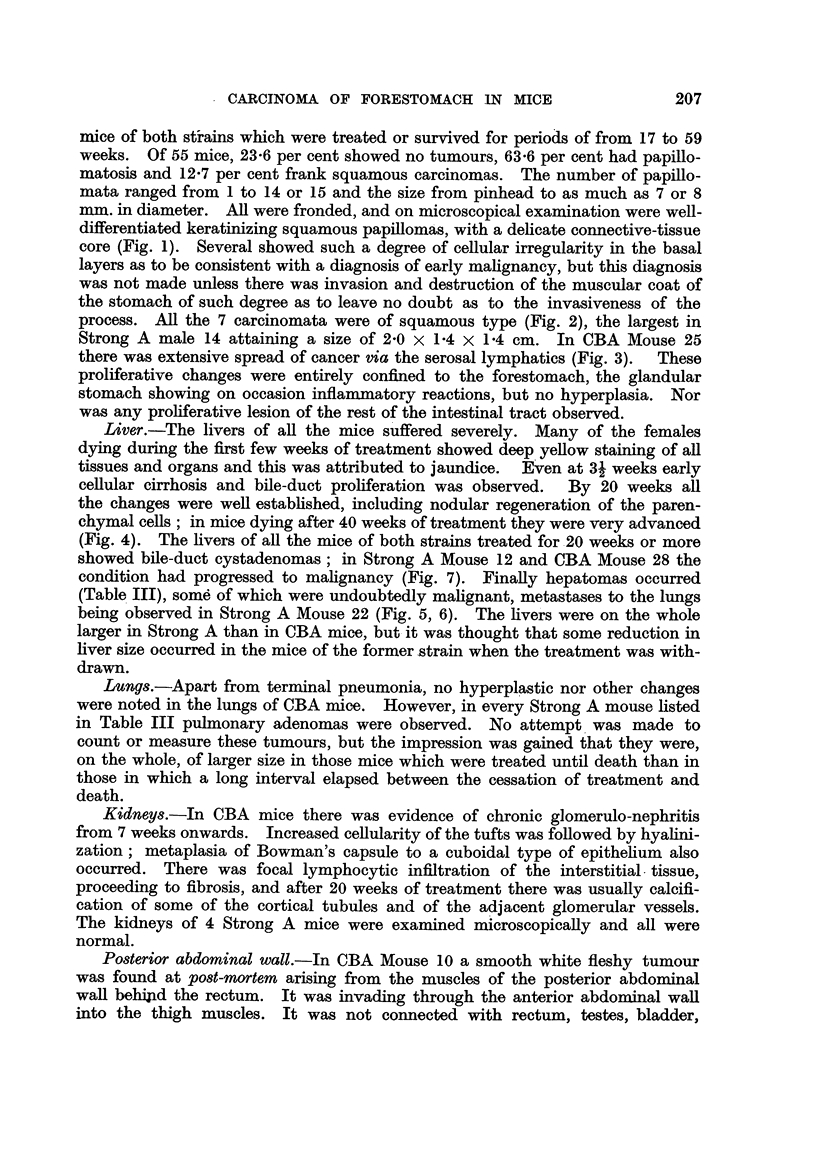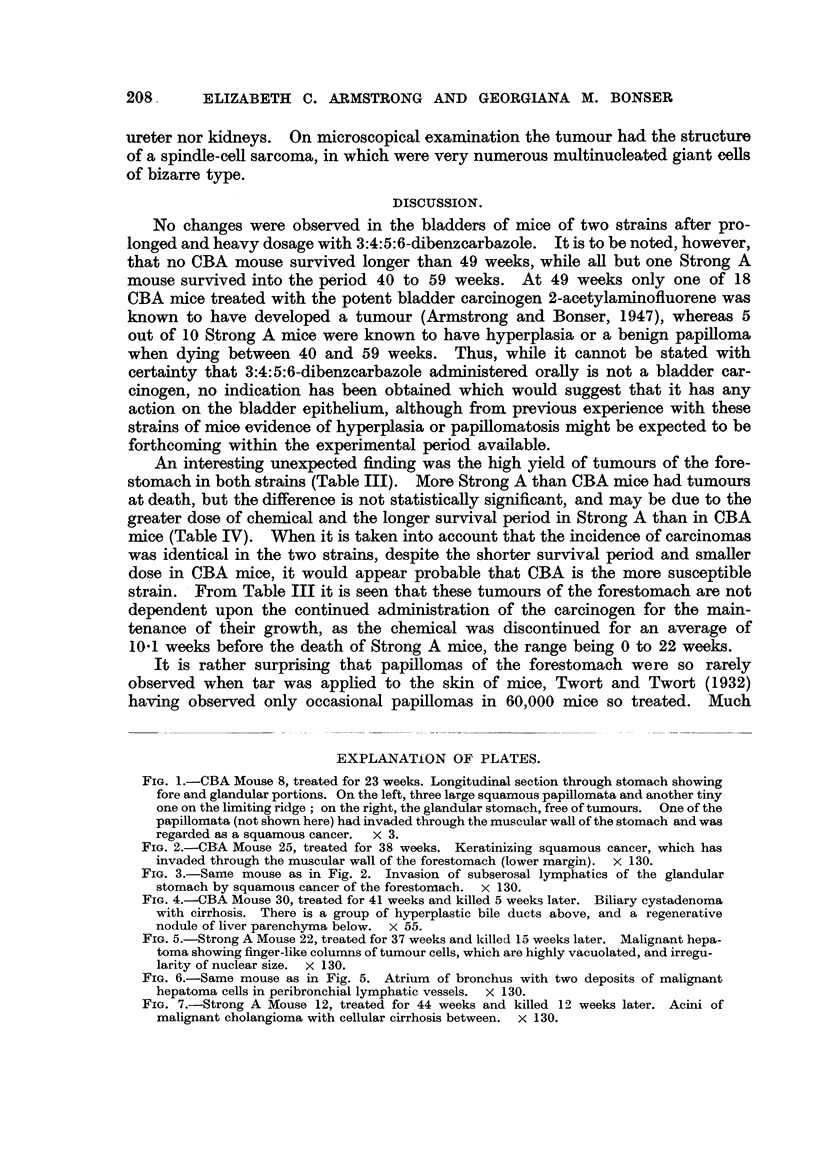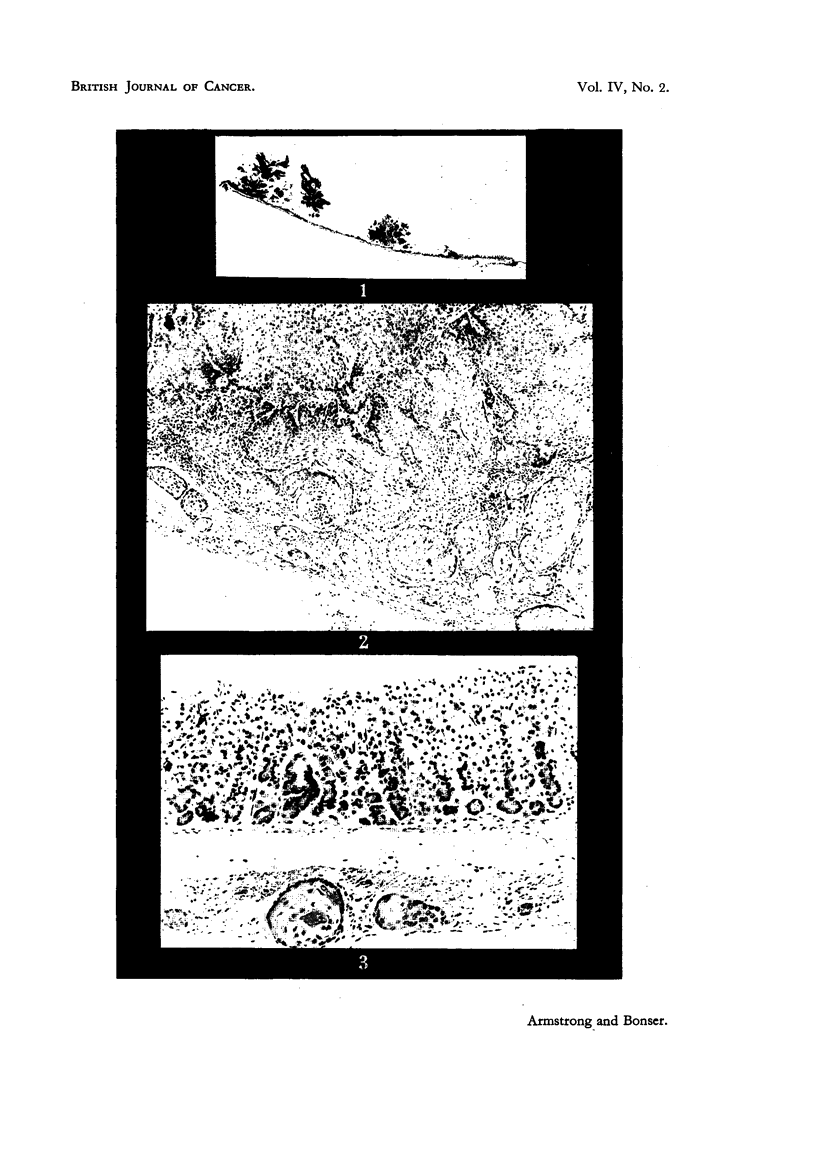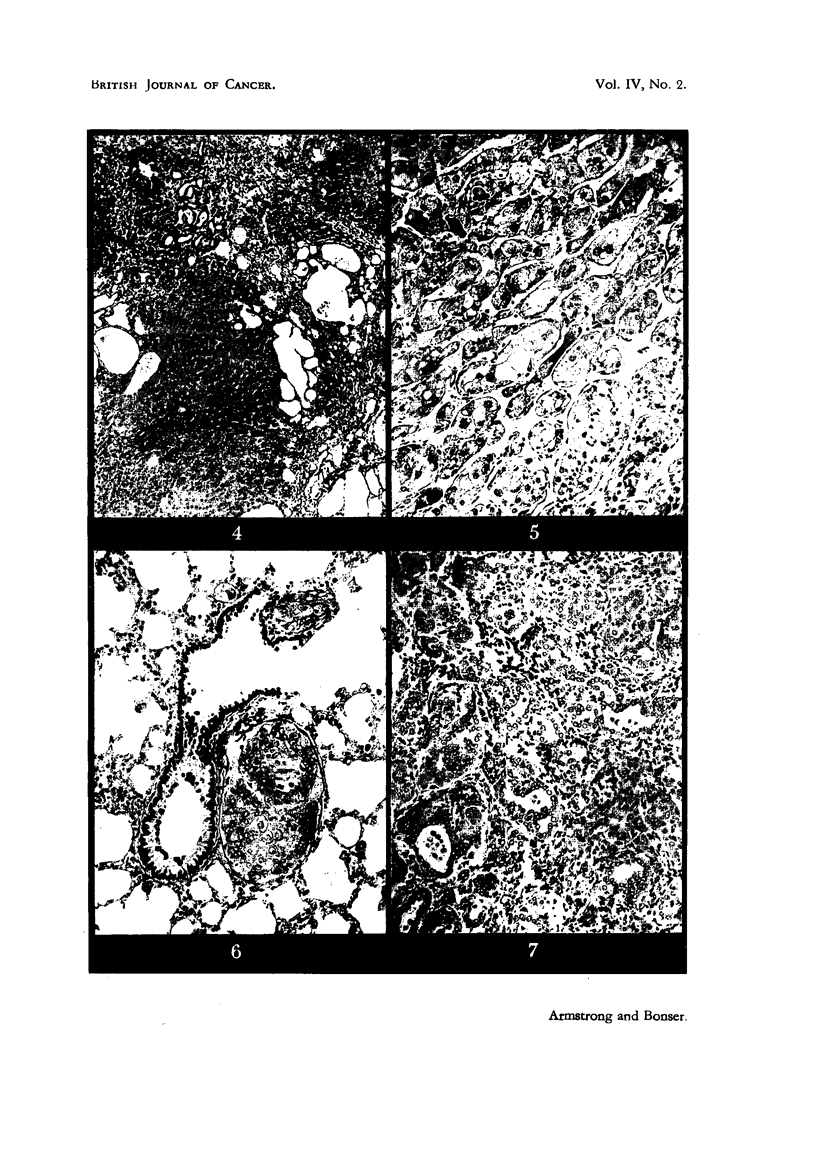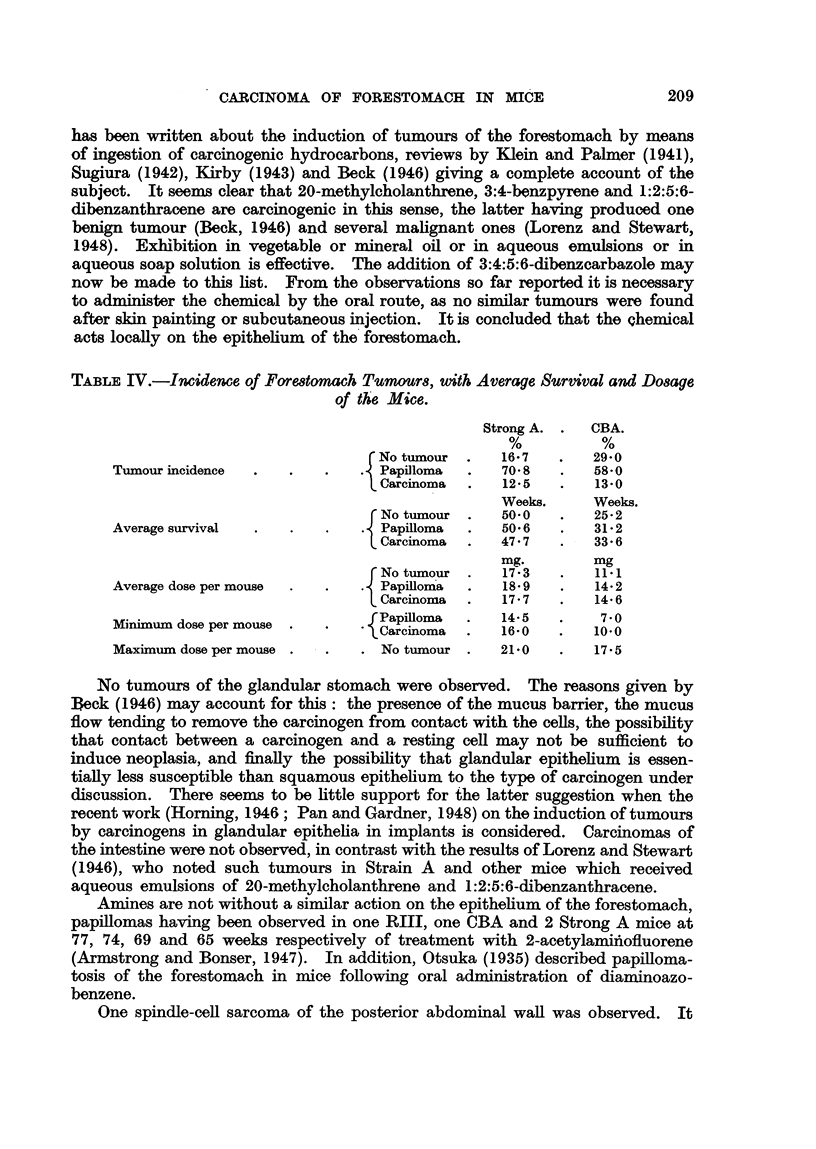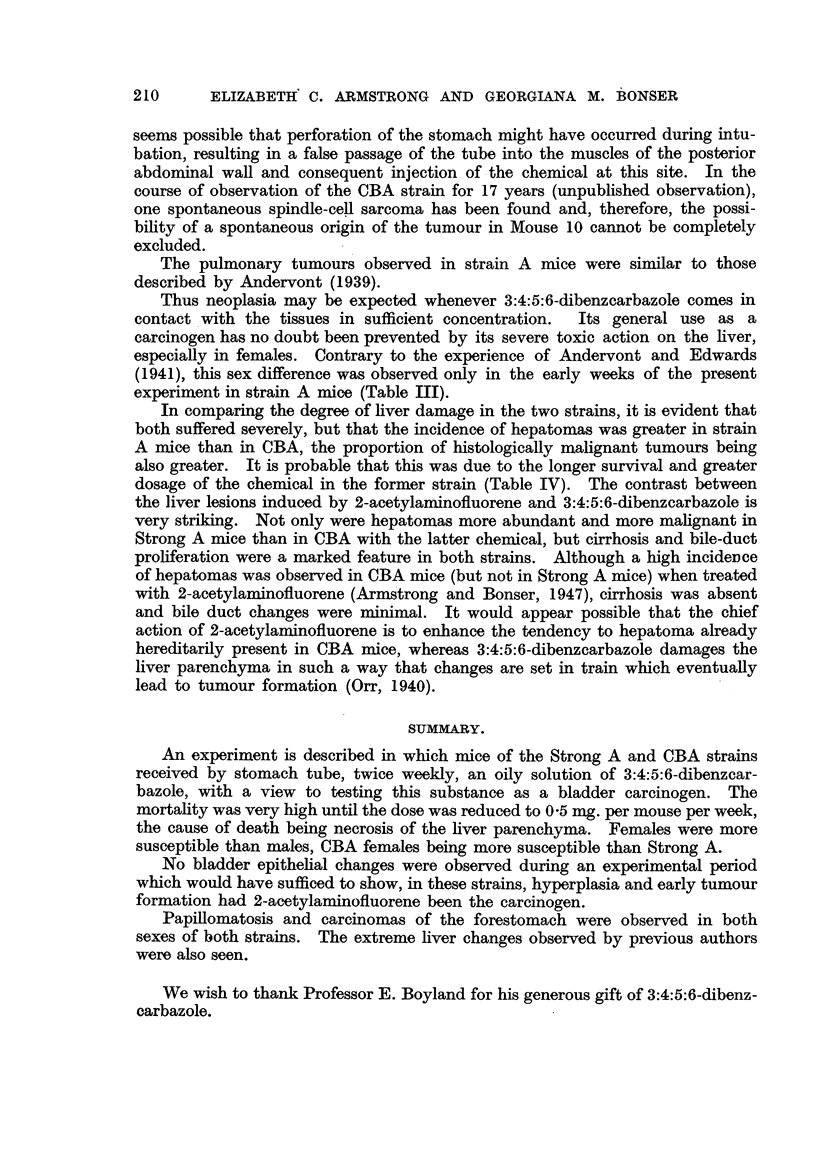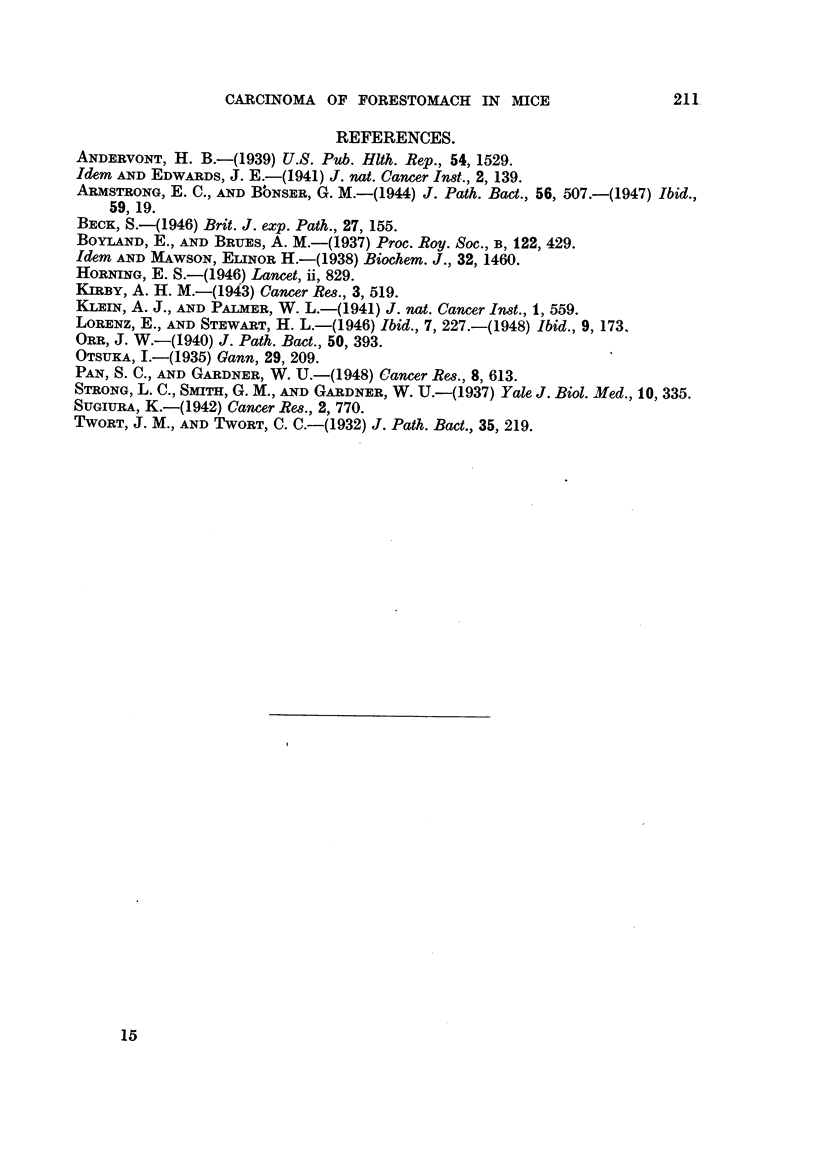# Squamous Carcinoma of the Forestomach and other Lesions in Mice following Oral Administration of 3:4:5:6-Dibenzcarbazole

**DOI:** 10.1038/bjc.1950.20

**Published:** 1950-06

**Authors:** Elizabeth C. Armstrong, Georgiana M. Bonser

## Abstract

**Images:**


### SQUAMOUS CARCINOMA OF THE FORESTOMACH AND OTHER LESIONS IN MICE FOLLOWING ORAL ADMINISTRATION OF 3:4:5:6-DIBENZCARBAZOLE.

ELIZABETH C. ARMSTRONG AND GEORGIANA M. BONSER.

*From the Department of Experimental Pathology and Cancer Research, University of Leeds.*

Received for publication March 13, 1950.

3:4:5:6-Dibenzcarbazole has been tested on numerous occasions and has been shown to be a potent carcinogen in mice (Boyland and Brues, 1937; Strong, Smith and Gardner, 1937; Boyland and Mawson, 1938; Andervont, 1939; Andervont and Edwards, 1941). The results are summarized in Table I. Benign and malignant cutaneous tumours were observed after skin painting and subcutaneous sarcomas after injection. In both types of experiment distant tumours were found in the lungs and liver. In the latter organ the changes were very severe and comprised cirrhosis and bile-duct proliferation, which proceeded to the formation of cholangiomas, as well as hepatomas.

As Boyland and Brues (1937) had suggested that naphthylamine workers might come in contact with dibenzcarbazoles during the course of their work, it was decided to re-investigate 3:4:5:6-dibenzcarbazole as a potential bladder carcinogen. For this purpose it seemed probable that much larger doses than those used hitherto would be required. Mice of the CBA strain were chosen, as this strain had previously given a higher yield of bladder tumours when 2-acetyl-aminofluorene was fed by stomach tube than four other strains tested (Armstrong and Bonser, 1947). Later, mice of the Strong A strain were also included, as the livers of these mice suffered the least damage in the above experiment.

### METHOD.

Mice of the two strains CBA and Strong A were used, having been brother-sister bred in the laboratory. At the age of 12 weeks administration of 3:4:5:6-dibenzcarbazole, supplied by Professor E. Boyland (Chester Beatty Research Institute, Royal Cancer Hospital, London), in arachis oil by stomach tube twice per week was commenced. The technique of this method of administration is described by Armstrong and Bonser (1944). As the mortality in CBA mice in the first few weeks was very high, successive groups were tested at different dosage until a reasonable survival was obtained (Table II). The CBA mice were treated until death; in Strong A mice, when it was evident from the swollen state of the abdomen that much liver damage had taken place, treatment was continued for as long as the mice seemed able to support it and was then withheld until death, the interval in some cases being over 20 weeks (Table III). By this means it was possible to prolong the life of both sexes into the period 50 to 60 weeks.

The bladder was inspected with the naked eye or with the dissecting microscope

TABLE I.—*Summary of Results of Previous Workers.*

| Author. | Mice tested. Number. | Type. | Sex. | Neoplasms observed. | Approximate time range. | Mode of administration. | Dose. (mg.) |
|---|---|---|---|---|---|---|---|
| Boyland and Brues (1937) | 26 | Stock mice, Simpson strain | .. | 4 papilloma skin, 4 carcinoma skin | 100–321 days | Skin painting (0·3% reduced to 0·1%) | 4·2 (approx.) 4·8 |
| | 25 | ,, | .. | 4 carcinoma skin, 5 hepatomas | 150–321 ,, | Skin painting (0·1%) | 2·4 6·2 |
| Strong, Smith and Gardner (1937) | 5 | CBA mice | ♂ | 4 carcinoma skin,* 1 spindle cell sarcoma | Several months | Skin painting (0·5%) | Not known |
| | 18 | ,, | ♂ | 14 subcutaneous sarcomas, 5 hepatomas, 2 pulmonary tumours | 204 days | 6 subcutaneous injections | 1·2 |
| | 24 | ,, | ♂ | 24 subcutaneous sarcomas, 3 hepatomas | 145 days | ,, | 1·2 |
| | 18 | A mice | ♂ | 17 subcutaneous sarcomas | 138 days | ,, | 1·2 |
| Boyland and Mawson (1938) | 28 | Stock mice | .. | 1 sarcoma at site of injection | 176 days | 1 intraperitoneal injection | 0·25 |
| | 83 | ,, | .. | 33 bile duct proliferation or cholangioma | 35–200 days | ,, | 0·25 |
| Andervont (1939) | 20 | A mice | ♀ | 78% pulmonary tumours | 4·2 months | 1 subcutaneous injection | 0·2 |
| Andervont and Edwards (1941) | 170 | ,, | ♀ | 45·4% subcutaneous sarcomas, 67·8% pulmonary tumours | 10–42 weeks ,, | 1 subcutaneous injection | 0·2 |
| | 83 | ,, | ♂ | 55·3% subcutaneous sarcomas | ,, | ,, | 0·2 |
| | 11 | C3H mice | ♀ | 45·5% ,, | ,, | ,, | 0·2 |
| | 49 | ,, | ♂ | 41·5% ,, | ,, | ,, | 0·2 |
| | 23 | C mice | ♀ | 47·8% ,, | ,, | ,, | 0·2 |
| | 44 | ,, | ♂ | 79·5% ,, | ,, | ,, | 0·2 |

* Including one carcino-sarcoma.

in all cases, previous experience having shown that it is not difficult to detect early bladder lesions by these means. A microscopical examination was also made in approximately one-quarter of the mice.

RESULTS.

*Mortality.*—In the early weeks the death-rate of CBA females in Experiments I to III was much greater than that of males (Table II). Even when, in Experiment III, the dose was reduced to 0·5 mg. per week, a level fairly well tolerated by the males (Experiment II*b*), all the females died before the expiration of 5 weeks. The cause of death was peripheral lobular liver cell necrosis, upon which pneumonia supervened. The dose was not found which would permit the females to live for more than 20 weeks, although 27 out of 60 CBA males survived from 20 to 49 weeks.

TABLE II.—*Five Experiments in which* 3:4:5:6-*dibenzcarbazole was Administered to CBA and Strong A Mice in Solution in Arachis Oil by Stomach Tube Twice Weekly.*

| Expt. | Number of mice. F. | Number of mice. M. | Treatment. | Dose per week mg. | Deaths. F. | Deaths. M. | Surviva (weeks.) |
|---|---|---|---|---|---|---|---|
| Ia | 13 | 15 | 0·2 c.c. of 1 per cent solution reduced after one week to 0·1 c.c. of 1 per cent solution | 4 | 11 | 1 | 1 |
| | | | | 2 | 2 | 14 | 3–12 |
| Ib | .. | 9 | 0·1 c.c. of 1 per cent solution reduced after 7 weeks to 0·1 c.c. of 0·5 per cent solution | 2 | 0 | 3 | 0–9 |
| | | | | 1 | 0 | 3 | 10–19 |
| | | | | | 0 | 3 | 20–39 |
| IIa | 9 | 11 | 0·1 c.c. of 0·5 per cent solution | 1 | 8 | 4 | 0–9 |
| | | | | | 0 | 1 | 10–19 |
| | | | | | 1 | 6 | 20–29 |
| IIb | .. | 10 | 0·1 c.c. of 0·5 per cent solution reduced at varying times to 0·1 c.c. of 0·25 per cent solution | 1 | 0 | 2 | 10–19 |
| | | | | | 0 | 4 | 20–29 |
| | | | | $\frac{1}{2}$ | 0 | 4 | 30–49 |
| III | 14 | .. | 0·1 c.c. of 0·25 per cent solution | $\frac{1}{2}$ | 14 | .. | 0–5 |
| IV | 13 | 15 | 0·1 c.c. of 0·125 per cent solution increased after 6 weeks to 0·1 c.c. of 0·25 per cent solution | $\frac{1}{4}$ | 9 | 2 | 0–9 |
| | | | | | 4 | 3 | 10–19 |
| | | | | $\frac{1}{2}$ | 0 | 2 | 20–29 |
| | | | | | 0 | 6 | 30–39 |
| | | | | | 0 | 2 | 40–49 |
| V | 18 | 12 | 0·1 c.c. of 0·125 per cent solution increased after 6 weeks to 0·1 c.c. of 0·25 per cent solution | $\frac{1}{4}$ | 5 | 1 | 0–9 |
| | | | | | 1 | 1 | 30–39 |
| | | | | $\frac{1}{2}$ | 1 | 6 | 40–49 |
| | | | | | 11 | 4 | 50–59 |

In experiments I to IV, CBA mice were used. In experiment V, Strong A mice were used.

In Experiment V, Strong A female mice showed only a slight tendency to die early from the effects of the chemical with the dose given, 5 out of 18 dying in the first few weeks, but when once tolerance had been established the females survived rather longer than the males (Table II).

*Bladder.*—No epithelial changes were observed.

*Forestomach.*—Squamous papillomata of the forestomach were observed in a CBA male mouse after 17 weeks of treatment. In Table III are listed all the

TABLE III.—*Occurrence of Gastric and Liver Tumours.*

**Strong A Mice.**

| Mouse number. | Sex. | Weeks of survival. | Total dose of chemical (mg.). | Stomach. No tumour. | Stomach. Pap. | Stomach. Carc. | Liver. No tumour. | Liver. Benign hep. | Liver. Mal. hep. |
|---|---|---|---|---|---|---|---|---|---|
| 1 | ♀ | 36 (35) | 16·0 | .. | + | .. | .. | + | .. |
| 2 | ♀ | 45 (32) | 14·5 | .. | + | .. | .. | .. | + |
| 3 | ♀ | 51 (35) | 16·0 | .. | .. | + | .. | .. | + |
| 4 | ♀ | 51 (35) | 16·0 | .. | + | .. | .. | .. | + |
| 5 | ♀ | 51 (35) | 16·0 | .. | + | .. | .. | .. | + |
| 6 | ♀ | 52 (31) | 14·0 | .. | + | .. | .. | .. | + |
| 7 | ♀ | 52 (31) | 14·0 | 0 | .. | .. | .. | .. | + |
| 8 | ♀ | 53 (32) | 14·5 | .. | + | .. | 0 | .. | .. |
| 9 | ♀ | 55 (44) | 20·5 | .. | + | .. | 0 | .. | .. |
| 10 | ♀ | 56 (44) | 20·5 | .. | + | .. | .. | .. | + |
| 11 | ♀ | 56 (44) | 20·5 | .. | + | .. | .. | .. | + |
| 12 | ♀ | 56 (44) | 20·5 | .. | + | .. | .. | + | .. |
| 13 | ♀ | 56 (44) | 20·5 | .. | .. | + | .. | .. | + |
| 14 | ♂ | 36 (36) | 16·5 | .. | .. | + | .. | + | .. |
| 15 | ♂ | 45 (45) | 21·0 | .. | + | .. | .. | .. | + |
| 16 | ♂ | 45 (45) | 21·0 | 0 | .. | .. | .. | .. | + |
| 17 | ♂ | 47 (43) | 20.0 | .. | + | .. | .. | .. | + |
| 18 | ♂ | 48 (44) | 20·5 | .. | + | .. | .. | + | .. |
| 19 | ♂ | 49 (49) | 23·0 | .. | + | .. | .. | .. | + |
| 20 | ♂ | 49 (49) | 23·0 | .. | + | .. | .. | .. | + |
| 21 | ♂ | 51 (37) | 17·0 | 0 | .. | .. | .. | .. | + |
| 22 | ♂ | 52 (37) | 17·0 | 0 | .. | .. | .. | .. | +† |
| 23 | ♂ | 52 (48) | 22·5 | .. | + | .. | .. | .. | + |
| 24 | ♂ | 59 (37) | 17·0 | .. | + | .. | .. | .. | + |
| 24 | 13 ♀ 11 ♂ | Av. = 50·1 (40·0) | Av. = 17·8 | 4 | 17 | 3 | 2 | 4 | 18 |

**CBA Mice.**

| Mouse number. | Sex. | Weeks of treatment. | Total dose of chemical (mg.). | Stomach. No tumour. | Stomach. Pap. | Stomach. Carc. | Liver. No tumour. | Liver. Benign hep. | Liver. Mal hep. |
|---|---|---|---|---|---|---|---|---|---|
| 1 | ♂ | 17 | 7·0 | .. | + | .. | .. | + | .. |
| 2 | ♂ | 19 | 8·0 | 0 | .. | .. | 0 | .. | .. |
| 3 | ♀ | 20 | 8·5 | .. | + | .. | 0 | .. | .. |
| 4 | ♂ | 20 | 8·5 | 0 | .. | .. | 0 | .. | .. |
| 5 | ♂ | 20 | 8·5 | 0 | .. | .. | 0 | .. | .. |
| 6 | ♂ | 21 | 9·0 | 0 | .. | .. | 0 | .. | .. |
| 7 | ♂ | 22 | 9·5 | 0 | .. | .. | 0 | .. | .. |
| 8 | ♂ | 23 | 10·0 | .. | .. | + | .. | .. | + |
| 9 | ♂ | 24 | 10·5 | .. | + | .. | 0 | .. | .. |
| 10* | ♂ | 25 | 11·0 | .. | + | .. | 0 | .. | .. |
| 11 | ♂ | 25 | 11·0 | 0 | .. | .. | 0 | .. | .. |
| 12 | ♂ | 25 | 11·0 | .. | + | .. | .. | + | .. |
| 13 | ♂ | 27 | 12·0 | .. | + | .. | .. | + | .. |
| 14 | ♂ | 27 | 12·0 | .. | .. | + | .. | + | .. |
| 15 | ♂ | 27 | 12·0 | .. | + | .. | .. | + | .. |
| 16 | ♂ | 29 | 13·0 | .. | + | .. | .. | + | .. |
| 17 | ♂ | 29 | 13·0 | 0 | .. | .. | 0 | .. | .. |
| 18 | ♂ | 30 | 13·5 | .. | + | .. | .. | + | .. |
| 19 | ♂ | 30 | 13·5 | .. | + | .. | 0 | .. | .. |
| 20 | ♂ | 30 | 13·5 | .. | + | .. | .. | + | .. |
| 21 | ♂ | 34 | 15·5 | .. | + | .. | .. | + | .. |
| 22 | ♂ | 34 | 15·5 | 0 | .. | .. | .. | + | .. |
| 23 | ♂ | 37 | 17·0 | .. | + | .. | .. | + | .. |
| 24 | ♂ | 37 | 17·0 | 0 | .. | .. | 0 | .. | .. |
| 25 | ♂ | 38 | 17·5 | .. | .. | + | .. | + | .. |
| 26 | ♂ | 38 | 17·5 | .. | + | .. | .. | + | .. |
| 27 | ♂ | 39 | 18·0 | .. | + | .. | 0 | .. | .. |
| 28 | ♂ | 39 | 18·0 | .. | + | .. | 0 | .. | .. |
| 29 | ♂ | 41 | 19·0 | .. | + | .. | .. | + | .. |
| 30 | ♂ | 46‡ | 19·0 | .. | .. | + | .. | + | .. |
| 31 | ♂ | 49 | 23·0 | .. | + | .. | 0 | .. | .. |
| 31 | 1 ♀ 30 ♂ | Av. = 29·9 | Av. = 13·3 | 9 | 18 | 4 | 15 | 15 | 1 |

Figures in brackets denote weeks of treatment.

Pap. = papilloma.

Carc. = carcinoma.

Hep. = hepatoma.

* This mouse had a pleomorphic sarcoma of the muscles of the posterior abdominal wall.

† There were secondary deposits in the lung from this tumour.

‡ This mouse survived for 5 weeks after the last treatment was administered at 41 weeks.

mice of both strains which were treated or survived for periods of from 17 to 59 weeks. Of 55 mice, 23·6 per cent showed no tumours, 63·6 per cent had papillomatosis and 12·7 per cent frank squamous carcinomas. The number of papillomata ranged from 1 to 14 or 15 and the size from pinhead to as much as 7 or 8 mm. in diameter. All were fronded, and on microscopical examination were well-differentiated keratinizing squamous papillomas, with a delicate connective-tissue core (Fig. 1). Several showed such a degree of cellular irregularity in the basal layers as to be consistent with a diagnosis of early malignancy, but this diagnosis was not made unless there was invasion and destruction of the muscular coat of the stomach of such degree as to leave no doubt as to the invasiveness of the process. All the 7 carcinomata were of squamous type (Fig. 2), the largest in Strong A male 14 attaining a size of 2·0 × 1·4 × 1·4 cm. In CBA Mouse 25 there was extensive spread of cancer *via* the serosal lymphatics (Fig. 3). These proliferative changes were entirely confined to the forestomach, the glandular stomach showing on occasion inflammatory reactions, but no hyperplasia. Nor was any proliferative lesion of the rest of the intestinal tract observed.

*Liver*.—The livers of all the mice suffered severely. Many of the females dying during the first few weeks of treatment showed deep yellow staining of all tissues and organs and this was attributed to jaundice. Even at 3½ weeks early cellular cirrhosis and bile-duct proliferation was observed. By 20 weeks all the changes were well established, including nodular regeneration of the parenchymal cells ; in mice dying after 40 weeks of treatment they were very advanced (Fig. 4). The livers of all the mice of both strains treated for 20 weeks or more showed bile-duct cystadenomas ; in Strong A Mouse 12 and CBA Mouse 28 the condition had progressed to malignancy (Fig. 7). Finally hepatomas occurred (Table III), some of which were undoubtedly malignant, metastases to the lungs being observed in Strong A Mouse 22 (Fig. 5, 6). The livers were on the whole larger in Strong A than in CBA mice, but it was thought that some reduction in liver size occurred in the mice of the former strain when the treatment was withdrawn.

*Lungs*.—Apart from terminal pneumonia, no hyperplastic nor other changes were noted in the lungs of CBA mice. However, in every Strong A mouse listed in Table III pulmonary adenomas were observed. No attempt was made to count or measure these tumours, but the impression was gained that they were, on the whole, of larger size in those mice which were treated until death than in those in which a long interval elapsed between the cessation of treatment and death.

*Kidneys*.—In CBA mice there was evidence of chronic glomerulo-nephritis from 7 weeks onwards. Increased cellularity of the tufts was followed by hyalinization ; metaplasia of Bowman's capsule to a cuboidal type of epithelium also occurred. There was focal lymphocytic infiltration of the interstitial tissue, proceeding to fibrosis, and after 20 weeks of treatment there was usually calcification of some of the cortical tubules and of the adjacent glomerular vessels. The kidneys of 4 Strong A mice were examined microscopically and all were normal.

*Posterior abdominal wall*.—In CBA Mouse 10 a smooth white fleshy tumour was found at *post-mortem* arising from the muscles of the posterior abdominal wall behind the rectum. It was invading through the anterior abdominal wall into the thigh muscles. It was not connected with rectum, testes, bladder, ureter nor kidneys. On microscopical examination the tumour had the structure of a spindle-cell sarcoma, in which were very numerous multinucleated giant cells of bizarre type.

### DISCUSSION.

No changes were observed in the bladders of mice of two strains after prolonged and heavy dosage with 3:4:5:6-dibenzcarbazole. It is to be noted, however, that no CBA mouse survived longer than 49 weeks, while all but one Strong A mouse survived into the period 40 to 59 weeks. At 49 weeks only one of 18 CBA mice treated with the potent bladder carcinogen 2-acetylaminofluorene was known to have developed a tumour (Armstrong and Bonser, 1947), whereas 5 out of 10 Strong A mice were known to have hyperplasia or a benign papilloma when dying between 40 and 59 weeks. Thus, while it cannot be stated with certainty that 3:4:5:6-dibenzcarbazole administered orally is not a bladder carcinogen, no indication has been obtained which would suggest that it has any action on the bladder epithelium, although from previous experience with these strains of mice evidence of hyperplasia or papillomatosis might be expected to be forthcoming within the experimental period available.

An interesting unexpected finding was the high yield of tumours of the forestomach in both strains (Table III). More Strong A than CBA mice had tumours at death, but the difference is not statistically significant, and may be due to the greater dose of chemical and the longer survival period in Strong A than in CBA mice (Table IV). When it is taken into account that the incidence of carcinomas was identical in the two strains, despite the shorter survival period and smaller dose in CBA mice, it would appear probable that CBA is the more susceptible strain. From Table III it is seen that these tumours of the forestomach are not dependent upon the continued administration of the carcinogen for the maintenance of their growth, as the chemical was discontinued for an average of 10·1 weeks before the death of Strong A mice, the range being 0 to 22 weeks.

It is rather surprising that papillomas of the forestomach were so rarely observed when tar was applied to the skin of mice, Twort and Twort (1932) having observed only occasional papillomas in 60,000 mice so treated. Much

---

### EXPLANATION OF PLATES.

FIG. 1.—CBA Mouse 8, treated for 23 weeks. Longitudinal section through stomach showing fore and glandular portions. On the left, three large squamous papillomata and another tiny one on the limiting ridge ; on the right, the glandular stomach, free of tumours. One of the papillomata (not shown here) had invaded through the muscular wall of the stomach and was regarded as a squamous cancer. × 3.

FIG. 2.—CBA Mouse 25, treated for 38 weeks. Keratinizing squamous cancer, which has invaded through the muscular wall of the forestomach (lower margin). × 130.

FIG. 3.—Same mouse as in Fig. 2. Invasion of subserosal lymphatics of the glandular stomach by squamous cancer of the forestomach. × 130.

FIG. 4.—CBA Mouse 30, treated for 41 weeks and killed 5 weeks later. Biliary cystadenoma with cirrhosis. There is a group of hyperplastic bile ducts above, and a regenerative nodule of liver parenchyma below. × 55.

FIG. 5.—Strong A Mouse 22, treated for 37 weeks and killed 15 weeks later. Malignant hepatoma showing finger-like columns of tumour cells, which are highly vacuolated, and irregularity of nuclear size. × 130.

FIG. 6.—Same mouse as in Fig. 5. Atrium of bronchus with two deposits of malignant hepatoma cells in peribronchial lymphatic vessels. × 130.

FIG. 7.—Strong A Mouse 12, treated for 44 weeks and killed 12 weeks later. Acini of malignant cholangioma with cellular cirrhosis between. × 130.

1

2

3

has been written about the induction of tumours of the forestomach by means of ingestion of carcinogenic hydrocarbons, reviews by Klein and Palmer (1941), Sugiura (1942), Kirby (1943) and Beck (1946) giving a complete account of the subject. It seems clear that 20-methylcholanthrene, 3:4-benzpyrene and 1:2:5:6-dibenzanthracene are carcinogenic in this sense, the latter having produced one benign tumour (Beck, 1946) and several malignant ones (Lorenz and Stewart, 1948). Exhibition in vegetable or mineral oil or in aqueous emulsions or in aqueous soap solution is effective. The addition of 3:4:5:6-dibenzcarbazole may now be made to this list. From the observations so far reported it is necessary to administer the chemical by the oral route, as no similar tumours were found after skin painting or subcutaneous injection. It is concluded that the chemical acts locally on the epithelium of the forestomach.

TABLE IV.—*Incidence of Forestomach Tumours, with Average Survival and Dosage of the Mice.*

| | | Strong A. | CBA. |
|---|---|---|---|
| | | % | % |
| Tumour incidence | No tumour | 16·7 | 29·0 |
| | Papilloma | 70·8 | 58·0 |
| | Carcinoma | 12·5 | 13·0 |
| | | Weeks. | Weeks. |
| Average survival | No tumour | 50·0 | 25·2 |
| | Papilloma | 50·6 | 31·2 |
| | Carcinoma | 47·7 | 33·6 |
| | | mg. | mg |
| Average dose per mouse | No tumour | 17·3 | 11·1 |
| | Papilloma | 18·9 | 14·2 |
| | Carcinoma | 17·7 | 14·6 |
| Minimum dose per mouse | Papilloma | 14·5 | 7·0 |
| | Carcinoma | 16·0 | 10·0 |
| Maximum dose per mouse | No tumour | 21·0 | 17·5 |

No tumours of the glandular stomach were observed. The reasons given by Beck (1946) may account for this : the presence of the mucus barrier, the mucus flow tending to remove the carcinogen from contact with the cells, the possibility that contact between a carcinogen and a resting cell may not be sufficient to induce neoplasia, and finally the possibility that glandular epithelium is essentially less susceptible than squamous epithelium to the type of carcinogen under discussion. There seems to be little support for the latter suggestion when the recent work (Horning, 1946 ; Pan and Gardner, 1948) on the induction of tumours by carcinogens in glandular epithelia in implants is considered. Carcinomas of the intestine were not observed, in contrast with the results of Lorenz and Stewart (1946), who noted such tumours in Strain A and other mice which received aqueous emulsions of 20-methylcholanthrene and 1:2:5:6-dibenzanthracene.

Amines are not without a similar action on the epithelium of the forestomach, papillomas having been observed in one RIII, one CBA and 2 Strong A mice at 77, 74, 69 and 65 weeks respectively of treatment with 2-acetylaminofluorene (Armstrong and Bonser, 1947). In addition, Otsuka (1935) described papillomatosis of the forestomach in mice following oral administration of diaminoazobenzene.

One spindle-cell sarcoma of the posterior abdominal wall was observed. It seems possible that perforation of the stomach might have occurred during intubation, resulting in a false passage of the tube into the muscles of the posterior abdominal wall and consequent injection of the chemical at this site. In the course of observation of the CBA strain for 17 years (unpublished observation), one spontaneous spindle-cell sarcoma has been found and, therefore, the possibility of a spontaneous origin of the tumour in Mouse 10 cannot be completely excluded.

The pulmonary tumours observed in strain A mice were similar to those described by Andervont (1939).

Thus neoplasia may be expected whenever 3:4:5:6-dibenzcarbazole comes in contact with the tissues in sufficient concentration. Its general use as a carcinogen has no doubt been prevented by its severe toxic action on the liver, especially in females. Contrary to the experience of Andervont and Edwards (1941), this sex difference was observed only in the early weeks of the present experiment in strain A mice (Table III).

In comparing the degree of liver damage in the two strains, it is evident that both suffered severely, but that the incidence of hepatomas was greater in strain A mice than in CBA, the proportion of histologically malignant tumours being also greater. It is probable that this was due to the longer survival and greater dosage of the chemical in the former strain (Table IV). The contrast between the liver lesions induced by 2-acetylaminofluorene and 3:4:5:6-dibenzcarbazole is very striking. Not only were hepatomas more abundant and more malignant in Strong A mice than in CBA with the latter chemical, but cirrhosis and bile-duct proliferation were a marked feature in both strains. Although a high incidence of hepatomas was observed in CBA mice (but not in Strong A mice) when treated with 2-acetylaminofluorene (Armstrong and Bonser, 1947), cirrhosis was absent and bile duct changes were minimal. It would appear possible that the chief action of 2-acetylaminofluorene is to enhance the tendency to hepatoma already hereditarily present in CBA mice, whereas 3:4:5:6-dibenzcarbazole damages the liver parenchyma in such a way that changes are set in train which eventually lead to tumour formation (Orr, 1940).

### SUMMARY.

An experiment is described in which mice of the Strong A and CBA strains received by stomach tube, twice weekly, an oily solution of 3:4:5:6-dibenzcarbazole, with a view to testing this substance as a bladder carcinogen. The mortality was very high until the dose was reduced to 0·5 mg. per mouse per week, the cause of death being necrosis of the liver parenchyma. Females were more susceptible than males, CBA females being more susceptible than Strong A.

No bladder epithelial changes were observed during an experimental period which would have sufficed to show, in these strains, hyperplasia and early tumour formation had 2-acetylaminofluorene been the carcinogen.

Papillomatosis and carcinomas of the forestomach were observed in both sexes of both strains. The extreme liver changes observed by previous authors were also seen.

We wish to thank Professor E. Boyland for his generous gift of 3:4:5:6-dibenzcarbazole.

### REFERENCES.

ANDERVONT, H. B.—(1939) *U.S. Pub. Hlth. Rep.*, **54**, 1529.
*Idem* AND EDWARDS, J. E.—(1941) *J. nat. Cancer Inst.*, **2**, 139.
ARMSTRONG, E. C., AND BONSER, G. M.—(1944) *J. Path. Bact.*, **56**, 507.—(1947) *Ibid.*, **59**, 19.
BECK, S.—(1946) *Brit. J. exp. Path.*, **27**, 155.
BOYLAND, E., AND BRUES, A. M.—(1937) *Proc. Roy. Soc.*, B, **122**, 429.
*Idem* AND MAWSON, ELINOR H.—(1938) *Biochem. J.*, **32**, 1460.
HORNING, E. S.—(1946) *Lancet*, ii, 829.
KIRBY, A. H. M.—(1943) *Cancer Res.*, **3**, 519.
KLEIN, A. J., AND PALMER, W. L.—(1941) *J. nat. Cancer Inst.*, **1**, 559.
LORENZ, E., AND STEWART, H. L.—(1946) *Ibid.*, **7**, 227.—(1948) *Ibid.*, **9**, 173.
ORR, J. W.—(1940) *J. Path. Bact.*, **50**, 393.
OTSUKA, I.—(1935) *Gann*, **29**, 209.
PAN, S. C., AND GARDNER, W. U.—(1948) *Cancer Res.*, **8**, 613.
STRONG, L. C., SMITH, G. M., AND GARDNER, W. U.—(1937) *Yale J. Biol. Med.*, **10**, 335.
SUGIURA, K.—(1942) *Cancer Res.*, **2**, 770.
TWORT, J. M., AND TWORT, C. C.—(1932) *J. Path. Bact.*, **35**, 219.